# Whole exome sequencing reveals concomitant mutations of multiple FA genes in individual Fanconi anemia patients

**DOI:** 10.1186/1755-8794-7-24

**Published:** 2014-05-15

**Authors:** Lixian Chang, Weiping Yuan, Huimin Zeng, Quanquan Zhou, Wei Wei, Jianfeng Zhou, Miaomiao Li, Xiaomin Wang, Mingjiang Xu, Fengchun Yang, Yungui Yang, Tao Cheng, Xiaofan Zhu

**Affiliations:** 1 State Key Laboratory of Experimental Hematology, Institute of Hematology and Blood Diseases Hospital, Chinese Academy of Medical Sciences and Peking Union Medical College, Tianjin, China; 2 Center for Stem Cell Medicine, Chinese Academy of Medical Sciences, Beijing, China; 3 Beijing Institute of Genomics, CAS, Beijing, China; 4 Herman B Wells Center for Pediatric Research, Indiana University School of Medicine, Indianapolis, IN, USA

**Keywords:** Fanconi anemia, Exome sequencing, DNA repair, Concomitant mutation

## Abstract

**Background:**

Fanconi anemia (FA) is a rare inherited genetic syndrome with highly variable clinical manifestations. Fifteen genetic subtypes of FA have been identified. Traditional complementation tests for grouping studies have been used generally in FA patients and in stepwise methods to identify the FA type, which can result in incomplete genetic information from FA patients.

**Methods:**

We diagnosed five pediatric patients with FA based on clinical manifestations, and we performed exome sequencing of peripheral blood specimens from these patients and their family members. The related sequencing data were then analyzed by bioinformatics, and the FANC gene mutations identified by exome sequencing were confirmed by PCR re-sequencing.

**Results:**

Homozygous and compound heterozygous mutations of FANC genes were identified in all of the patients. The FA subtypes of the patients included FANCA, FANCM and FANCD2. Interestingly, four FA patients harbored multiple mutations in at least two FA genes, and some of these mutations have not been previously reported. These patients’ clinical manifestations were vastly different from each other, as were their treatment responses to androstanazol and prednisone. This finding suggests that heterozygous mutation(s) in FA genes could also have diverse biological and/or pathophysiological effects on FA patients or FA gene carriers. Interestingly, we were not able to identify *de novo* mutations in the genes implicated in DNA repair pathways when the sequencing data of patients were compared with those of their parents.

**Conclusions:**

Our results indicate that Chinese FA patients and carriers might have higher and more complex mutation rates in FANC genes than have been conventionally recognized. Testing of the fifteen FANC genes in FA patients and their family members should be a regular clinical practice to determine the optimal care for the individual patient, to counsel the family and to obtain a better understanding of FA pathophysiology.

## Background

Fanconi anemia (FA) is a rare inherited genetic syndrome with diverse clinical manifestations, including developmental defects, short stature, bone marrow failure, and a high risk of malignancies. The prevalence of FA is 1–5 per 1 million population, and the heterozygous carrier frequency is estimated at 1 in 300 persons [1]. Ninety percent of the patients experience bone marrow failure by the age of 40 years old [2]. While androgens and hematopoietic growth factors are initially effective in treating FA, the disease has a very poor prognosis that often leaves bone marrow transplantation as the only option for a cure, although gene therapy could be a potential treatment [2]. The clinical manifestations and treatment responses of FA patients are highly variable, likely due to multiple factors that are still not quite clear. The FA diagnosis is usually confirmed by a positive chromosomal breakage test (DEB test) and by the subtyping of FA (determination of the complementation group) [3,4].

To date, 15 genetic subtypes of FA have been identified [5-9]. An FA patient carries either homologous mutations on two of the same alleles or compound heterozygous mutations on two different alleles of one FA gene. The traditional complementation test for group studies has been used mostly in FA patients, rather than in their families, using a stepwise method [10,11]. If homologous or compound heterozygous mutations are found in an FA gene, the subtype of FA is then determined without knowing whether other FA genes might also be mutated in the same patient. Thus, while useful, this method could lead to incomplete genetic information for FA patients. More complex changes in FA genes in the same patient are plausible, as different subtypes of FA patients can have different clinical manifestations. Moreover, the complete genetic information of FA patients and their families regarding FA genes could potentially be valuable to the patients’ prognoses and treatment options, enabling prenatal DNA testing, permitting pre-implantation genetic diagnosis (PGD) for future pregnancies and excluding FA carriers from bone marrow donation and gene therapy. To obtain a comprehensive genomic picture of the FA genes in patients, we captured and sequenced the exomes of five FA families through peripheral blood (PB) specimens.

## Methods

### Diagnosis of FA, patients’ sample collection and genomic DNA isolation

All of the FA patients were outpatients. The diagnosis of FA was based on the clinical manifestations of the patients, MMC testing and single-cell gel electrophoresis tests using blood lymphocytes. Subsequent studies were approved by the ethics committee of the Institute of Hematology, CAMS/PUMC (KT2010072302). A total of five FA patients (designated Fa-001 to Fa-005) and their parents were recruited for this study and signed informed consent forms. One patient was later identified as an adopted child of the family (Fa-005). Peripheral blood (PB) samples from all of the subjects were collected after they were informed of the studies and had signed an institutional consent form and we also received consent from patients to publish the images and data from these samples. Genomic DNA from the FA patients and their parents were obtained from mononuclear cells for exome sequencing.

### Exome sequencing, data analysis and PCR re-sequencing

The genomic DNA samples were randomly fragmented by Covaris, and the DNA fragments with base pair peaks were approximately 150 to 200 bp long. Adapters were then ligated to both ends of the resulting fragments. The adapter-ligated templates were purified using Agencourt AMPure SPRI beads, and fragments with an insert size of approximately 250 bp were excised. The extracted DNA was amplified by ligation-mediated PCR (LM-PCR) and was purified and hybridized to the SureSelect Biotinylated RNA Library (BAITS, Agilent Inc.) for enrichment. Hybridized fragments were bound to streptavidin beads, whereas non-hybridized fragments were washed out after 24 h. Captured LM-PCR products were analyzed using an Agilent 2100 Bioanalyzer to estimate the magnitude of their enrichment. Each captured library was then loaded onto a Hiseq2000 Platform, and massive parallel sequencing was performed for each captured library independently to ensure that each sample had at least 50-fold coverage. Raw image files were processed by Illumina Pipeline software, version 1.6, for base-calling with default parameters, and the sequences of each individual were generated as 90 bp paired-end reads. The bioinformatics analyses (Additional file 1: Figure S1) were performed by BGI at Shenzhen, China. The mutations in the FANC genes identified by exome sequencing were further verified by traditional PCR. The SRA accession number for the Exome-seq data reported in this paper was SRA067806.

## Results

### Diagnosis of FA patients

**Figure 1:**
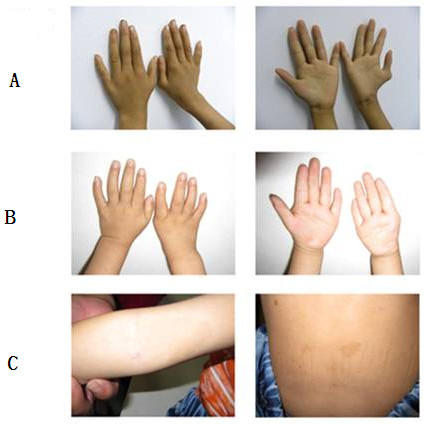
**Clinical manifestations of representative FA patients. A**: Hexadactylism; **B**: Absence of right hand thumb; **C**: Café au lait spots.

**Figure 2:**
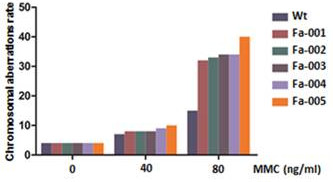
**Mitomycin C chromosome fragility test.** Mitomycin C chromosome fragility test of all of the FA patients and the normal controls (WT) at various MMC concentrations (count 100 cells).The patients’ chromosome fragility rates were higher than those of the controls, which indicated that the patients’ cells were MMC sensitive.

**Figure 3:**
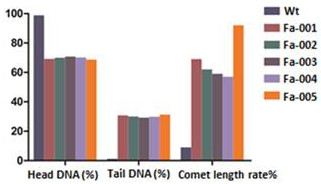
**Single-cell gel electrophoresis tests.** Single-cell gel electrophoresis test of all of the FA patients and the normal controls (WT) showed that the comet length rates of the patients were higher than those of the controls.

The clinical manifestations (Table 1 and Figure 1), MMC test results (Additional file 2: Table S1 and Figure 2) and single-cell gel electrophoresis test results (Additional file 3: Table S2 and Figure 3) are listed. The clinical manifestations of FA were variable but typically included hand abnormalities (Figure 1-A and B), café au lait spots (Figure 1-C), short stature, facial feature abnormalities and bone marrow failure in all of the patients. At an 80 ng/ml concentration of MMC, sample cells from these five patients showed significantly greater chromosomal abnormalities than normal controls. In the single-cell gel electrophoresis test, all five patients had significantly higher comet cell rates than the normal controls. The karyotypes of these five patients were normal (Additional file 4: Table S3). These findings supported the diagnosis of FA in the 5 patients [12].

**Table 1:** Clinical manifestation of FA patients

	**Fa-001**	**Fa-002**	**Fa-003**	**Fa-004**	**Fa-005**
**Sex**	Male	Male	Male	Female	Female
**Age at diagnosis (years)**	5	4	7	5	10
**History (months)**	18	18	84	5	108
**Café au lait spots**	Yes	Yes	No	No	Yes
**Short stature**	No	Yes	No	Yes	Yes
**Hand abnormalities**	Absence of right hand thumb. Radial eversion of left hand thumb.	No	Right hand thumb deformity. Hypoplastic thenar eminence of right hand.	Hexadactylism of right hand	Hexadactylism
**Facial features**	Microcephaly small eyes	No	No	No	Microcephaly
**Gonads**	No	Hypospadias	No	No	No
**Gastrointestinal tract abnormalities**	No	No	No	No	Yes
**Hematology 1 WBC**	2.09 × 10^9^/L	4.27 × 10^9^/L	2.67 × 10^9^/L	6.86 × 109/L	2.78 × 10^9^/L
**HGB**	32 g/L	93 g/L	78 g/L	106 g/L	57 g/L
**PLT**	9 × 10^9^/L	30 × 10^9^/L	14 × 10^9^/L	33 × 10^9^/L	42 × 10^9^/L
MMC (80 ng/ml)*	32	33	34	34	40
**Abnormal cell rate (%)**					
Single-cell gel electrophoresis test**	69	62	59	57	92
**Comet cell rates (%)**					
**Treatment**	**Androstanazol Prednisone**	**Androstanazol Prednisone**	**Androstanazol Prednisone**	**Traditional Chinese medicine**	**Androstanazol Prednisone**
**Blood transfusion**	4 U RBC/year	6 U RBC/year	No	No	No
	2 dose PLT/year	2 dose PLT/year			
**Hematology 2 WBC**	4.4 × 10^9^/L	3.55 × 10^9^/L	3.03 × 10^9^/L	4.7 × 10^9^/L	3.05 × 10^9^/L
**HGB**	70 g/L	78 g/L	101 g/L	71 g/L	86 g/L
**PLT**	16 × 10^9^/L	19 × 10^9^/L	105 × 10^9^/L	25 × 10^9^/L	75 × 10^9^/L

*Hematology 1*: The first time the patient was admitted to our hospital; *Hematology 2*: The day after treatment for 24 months ended.

* MMC tests: at an 80 ng/ml concentration of MMC, the percentage of cells with abnormal chromosomes (including chromosome breakage) in the controls was 15% (abnormality cell). The results showed that all of the patients had a greater percentage of abnormality cells than the controls.

** Single-cell gel electrophoresis tests, the comet cell rates of the controls was <10%. The results showed that all of the patients had significantly higher comet cell rates than the normal controls.

### Exome sequencing data analysis

Exome sequencing was performed by exome capture with an Agilent SureSelect Human All Exon Kit, the target region probes of which could cover is 37.2 Mb, and then was massively parallel paired-end 90 bp read sequenced; finally, we obtained 3.64 Gb raw bases on average for each sample. After mapping to the human reference genome (NCBI Build 36.3, hg 18), the target exome sequences had mean coverage of greater than 50-fold, on average, approximately 80% of the exomes were covered at least 20-fold, and approximately 90% of the exomes achieved at least 10-fold, with the coverage rate of the target region greater than 99%. After an initial analysis of the sequencing data of all of the FA genes, all of the identified mutations were re-sequenced for confirmation by traditional PCR (Additional file 5: Figure S2), the MAF of FANC gene SNPs, the SIFT and Polyphen prediction of the missense alterations are described in Additional file 6: Table S4. We found that compound heterozygous mutations represented the majority of the mutation types in all of the patients. The types of genetic aberrations found in this study included single nucleotide substitutions and small (1–8 nucleotides) deletions (Table 2 and Additional file 5: Figure S2). The mutations included SNPs reported in the NCBI database (the clinical significance of these mutations is unknown) and some novel mutations (Table 2). According to the mutation results and the results of other experiments, we identified the subtypes in all of our patients. The FA subtypes of these patients were FANCA (FA-001 FA-002 and FA-005), FANCM (FA-003) and FANCD2 (FA-004). FA001 was an FA-A patient with different mutations in each allele (one inherited from his father and the other from his mother). He had two mutations in the *FANCA* gene, Chr1688389826-32delGGGCTGT and Chr16883853-54delGG, which were not reported by the Rockefeller University Fanconi anemia mutation database, but Chr1688389829-30delCT has been reported 3 times, Chr16 88385351-54delGAGG has been reported once, and Chr16 88385351delG has been reported three times in the database, so we diagnosed Fa-001 with the subtype of FANCA. Interestingly, the boy patient FA-002 had concomitant homozygous mutations on A, B or D1. Based on sequencing results, FANCA was a possible cause of FA in this patient because he had a homozygous mutation. His mother had the same mutation, while his father did not. DNA sequencing results derived from oral epithelium cells confirmed the Chr16 88385436 position, G > A missense A > V mutation was indeed the same mutation as in the PB sample, indicating that the other copy of the mutation was congenital and did not come from his father. Fa-002 thus had a possible FA subtype. In contrast, *FANCB* was on the X chromosome, so it is possible that patient Fa-002 had a FANCB subtype. Our PCR re-sequencing confirmed that patient Fa-002 also has a homozygous mutation in the *FANCD1 (BRCA2)* gene in the 3’UTR region (see Table 2) and this position has been reported as an SNP site. This 3’UTR mutation is possible to be responsible for Fa-002’s condition since there were reports that some mutations in intron and UTR can also cause disease [13]. Thus we performed the complementation group testing and the patient was confirmed as the FANCA subtype. FA003 was an FANCM patient with different mutations in each allele (one from the father and the other from the mother). FA-004 was an FANCD2 patient with three different mutations in two alleles (one from the father and two from the mother). FA005, an adopted child with no parental genetic information, clearly had FANCA, because only three different mutations were identified among the *FANCA* genes. Surprisingly, we found that, except for patient Fa-005, all of the patients had multiple mutations in at least 2 FA genes (Table 2), consistent with what Settara C. Chandrasekharappa reported [13]. Although some mutations we found are the SNPs listed in the database, the functions of these mutations remain undetermined. Moreover, some SNPs can also cause disease [14], and thus we could not exclude that these SNPs are not related to FA disease. Finally, we were unable to detect new mutations in genes implicated in DNA repair pathways in any FA patients by comparison with their parents’ sequencing data.

**Table 2:** Validated Fanconi gene mutations

**Gene**	**Site**	**Mutation**	**Fa-001**			**Fa-002**			**Fa-003**			**Fa-004**			**Fa-005**
			**F**	**M**	**C**	**F**	**M**	**C**	**F**	**M**	**C**	**F**	**M**	**C**	**C**^**#**^
**FANCA**	Chr16 88389826-32	-GGGCTGT deletion frameshift											**√**	**√**	
	Chr16 88385436*	G > A missense A > V					**√**	**※**							
	Chr16 88389853-4	-GG deletion frameshift	**√**		**√**										
	Chr16 88385373	-A deletion frameshift		**√**	**√**										
	Chr16 88343697*	T > C missense S > G					**√**	**√**							
	Chr16 88343715	G > A missense R > W													**√**
	Chr16 88367267*	G > C missense P > A													**√**
	Chr16 88343815*	A > G intron													**√**
**FANCB**	ChrX 14781120	C > T missense V > I					**√**	**※**							
**FANCM**	Chr14 44714456	A > G missense I- > V								**√**	**√**				
	Chr14 44735218	C > G missense P > A				**√**		**√**							
	Chr14 44714339	G > T nonsense N > N				**√**		**√**							
	Chr14 44676037	C > T missense S > F				**√**		**√**							
	Chr14 44720650	A > G missense I > V				**√**		**√**							
	Chr14 44727906	G > A missense R > Q							**√**		**√**				
**FANCD1 (BRCA2)**	Chr13 31809499	C > G missense H > D										**√**		**√**	
	Chr13 31804480*	A > C missense N > H								**√**	**√**				
	Chr13 31809463*	A > C missense N > D								**√**	**√**				
	Chr13 31871012	A > C 3’-UTR				**√**	**√**	**※**							
**FANCD2**	Chr3 10117949*	C > T 3’-UTR										**√**		**√**	
	Chr3 10081532*	C > T missense P > L											**√**	**√**	
	Chr3 10115671*	G > A 3’-UTR											**√**	**√**	
**FANCI**	Chr15 87636941*	A > G missense I > V	**√**		**√**										

*※*:homozygous mutation; *√*: heterozygous mutation; *F*: father; *M*: mother; *C*: child (patient); *#* Orphan; *SNP.

## Discussion

FA is a recessive inherited disease with FA gene mutations that are primarily involved in DNA damage response or repair, resulting in genomic instability [13]. Its complex clinical manifestations are associated with different FA subtypes. Interestingly, the clinical manifestations can also differ among patients with the same FA subtype. There is no clear explanation for the relationship between the clinical manifestations of FA patients and the genetic mutations in their FA genes. To this end, we performed exome sequencing on PB samples from 5 FA patients and from their parents. Although the sample size in our study was small, the high frequency of heterozygous mutations in 4 of 5 patients, compared with their biological parents, suggested that the mutational events in FA carriers might not occur randomly but rather are linked. It is probable that a core determinant for some, if not all, FA genes governs the susceptibility of FA pathway gene mutations and, thus, of FA clinical manifestations.

In clinical practice, complementation group assignment and mutational analysis have been routinely used to identify types of FA patients, with the latter becoming more prevalent in recent years. The usual strategy for mutation analysis is to sequence the *FANCA, FANCC, FANCE, FANCG, FANCD2* and other FA genes sequentially until a subtype of FA is identified [10,11]. The correct subtyping of FA patients is critical for their prognoses and treatment because of the clinical variability among subtypes [1,15,16]. It is conceivable that the specific mutation theory is not always reflected in FA phenotypes because siblings with identical mutations can have different FA phenotypes [17]. Heterozygous mutations in FA genes can also have diverse biological and/or pathophysiological effects on FA patients or FA gene carriers [18-25]. This finding is also in agreement with our study, in which four patients had more than one FA mutation gene with, vastly distinct clinical manifestations and different treatment responses to androstanazol and prednisone. Furthermore, it should be noted that synonymous mutations and known SNPs can also contribute to FA, although each of these mutations must be studied individually. It will be interesting to determine whether the diverse clinical manifestations, cancer susceptibilities and biological properties in the same or different FA subtypes reflect specific combinations of multiple heterozygous FA gene mutations. Here, we suggest that all FA genes should be subjected to mutation analysis in clinically diagnosed FA patients and their parents, in addition to complementation group analysis, to ascertain the most accurate diagnosis of the FA subtype, to aid clinicians, as well as families, during genetic counseling process and to advance FA research and future gene and stem cell therapies.

Although the sample size of our study was small, the striking number of concomitant mutations in the FA genes (excluding the SNPs) lead us to believe that sequencing of large samples of FA patients and their families, as well as more detailed biological and functional studies of these samples, are needed to ascertain the relevance of heterozygous FA gene mutation(s) to the clinical manifestations and prognoses of FA patients.

To our surprise, we did not find any new mutations related to DNA repair pathway genes, especially given that FA is considered to be a genomic instability disorder. There are several possible explanations that could account for this negative finding. First, new mutations in DNA repair pathways in HSCs might occur at low frequencies such that those HSCs with new somatic mutations did not have a growth advantage at the time of the sample collection. Second, some epigenetic mechanisms other than additional genetic changes, such as mutations, could be involved in the pathogenic process [26,27]. Third, it is also possible that some of the mutated genes detected by exome-seq might have participated in DNA repair, but they are currently unknown. Nevertheless, the sequence analysis of our current data set suggested that mutations in the FA genes were sufficient for the manifestations of FA patients. Further studies are needed to ascertain this observation with larger FA patient pool sizes, to compare the exome sequencing data of patients before and after the development of cancer.

## Conclusions

In conclusion, although FA subtype mutations define an FA patient, we found that multiple heterozygous FA gene mutations inherited from the parents could occur concomitantly in the same FA patient. The higher mutation rate among FA genes in the same patient also hinted that a common upstream event could determine the mutational susceptibility of the FA pathway, and mutations in FA genes alone might be sufficient to initiate FA in the hematopoietic system. Therefore, our current report has important implications for the pathogenesis of FA, as well as for the clinical management (such as treatment options) of FA patients.

## Competing interests

The authors declare that they have no competing interests.

## Authors’ contributions

LXC and WPY performed the experiments and/or analyzed the results with the assistance of HMZ, QQZ, WW, JFZ, XMW, MML, FCY, MJX and YGY; WPY, LXC, TC and XFZ designed the research, analyzed the results and/or wrote the paper. All authors declare and approved the final manuscript.

## Pre-publication history

The pre-publication history for this paper can be accessed here:

http://www.biomedcentral.com/1755-8794/7/24/prepub

## Supplementary Material

Additional file 1: Figure S1 The bioinformatics analysis flow chart. Click here for file

Additional file 2: Table S1 Mitomycin C chromosome fragility test results. Click here for file

Additional file 3: Table S2 Single-cell gel electrophoresis test results. Click here for file

Additional file 4: Table S3 Karyotype and bone marrow cellularity of FA patients. Click here for file

Additional file 5: Figure S2 FA gene re-sequencing results of the FA patients and their parents. Click here for file

Additional file 6: Table S4 MAF, SIFT and Polyphen-2 analysis of FA mutations. Click here for file

## Accession Number

The NCBI accession number for the exome sequence reported in this paper is SRA067806.
