# Supplementary figures and images for: Whole exome sequencing reveals concomitant mutations of multiple FA genes in individual Fanconi anemia patients
Source: BMC Med Genomics. 2014 May 15;7:24. doi: 10.1186/1755-8794-7-24 (PMC4038598; doi:10.1186/1755-8794-7-24)

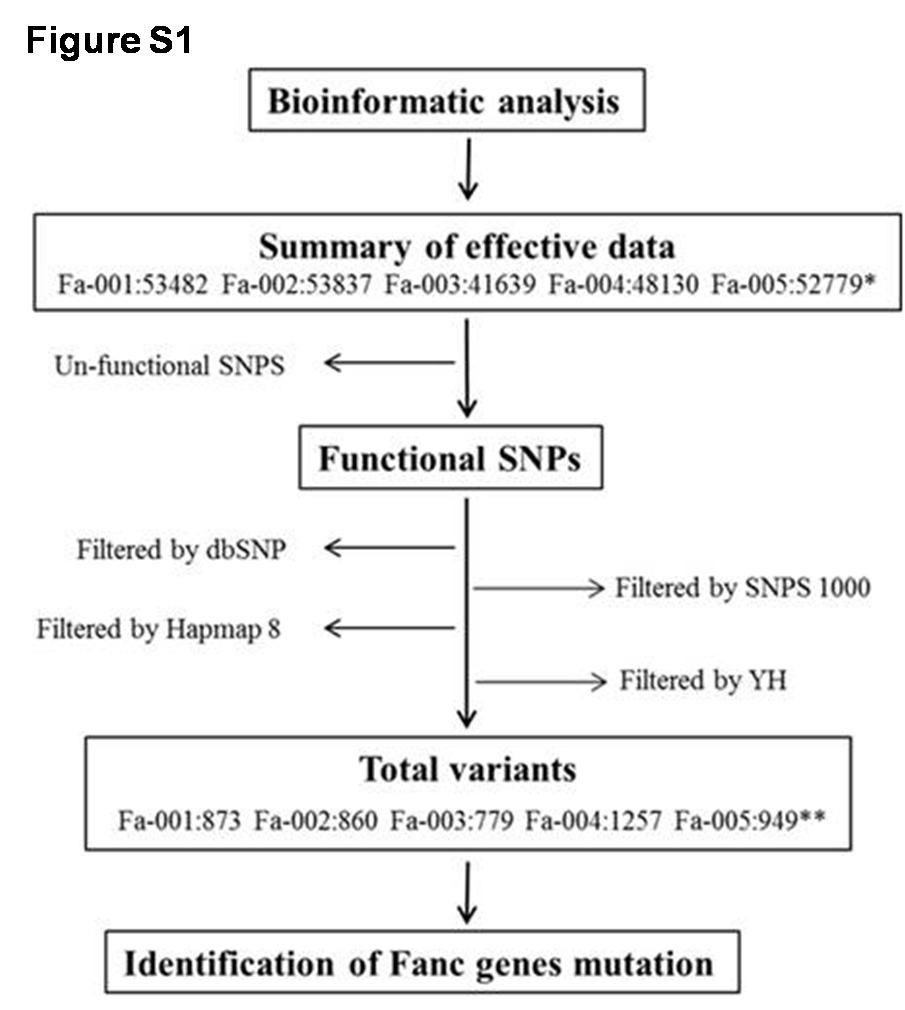

Supplement: Additional file 1: Figure S1 — The bioinformatics analysis flow chart. [file 1755-8794-7-24-S1.tiff]

**Figure S2. FA gene re-sequencing results of FA patients and their parents.**

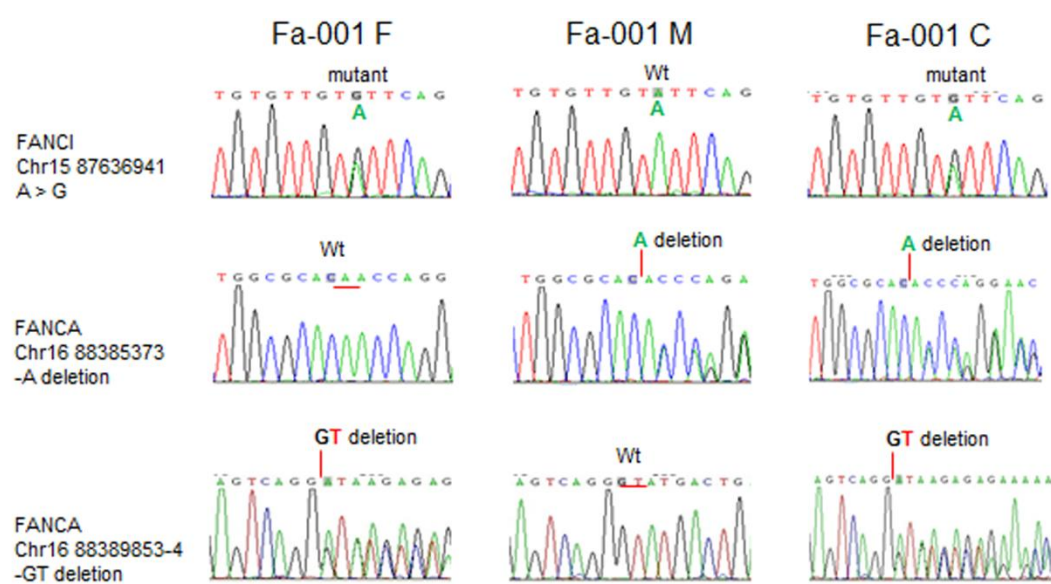

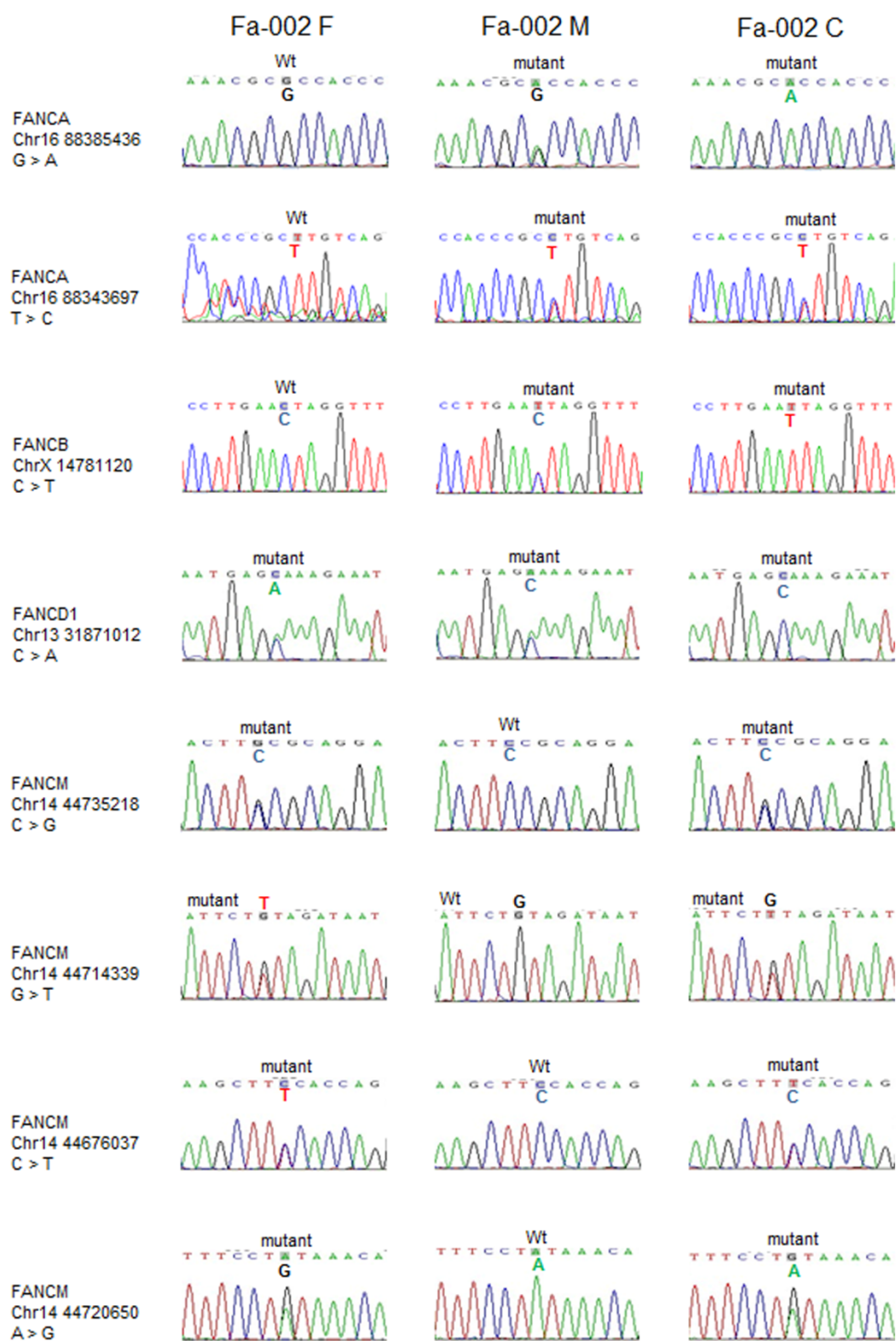

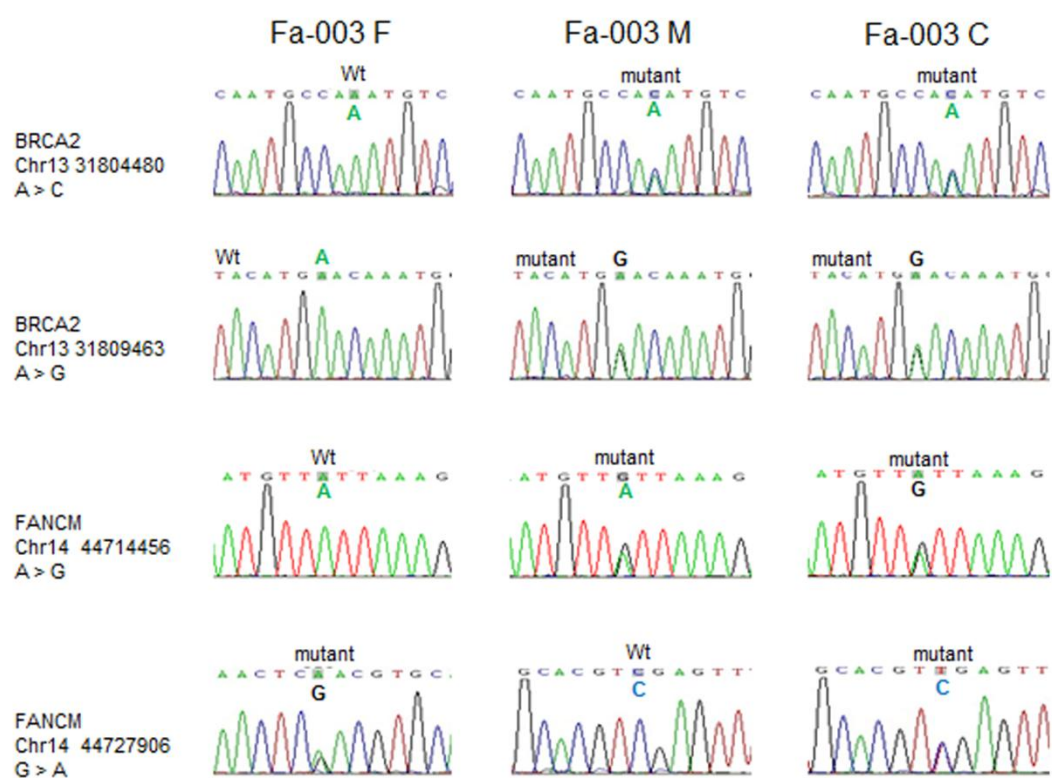

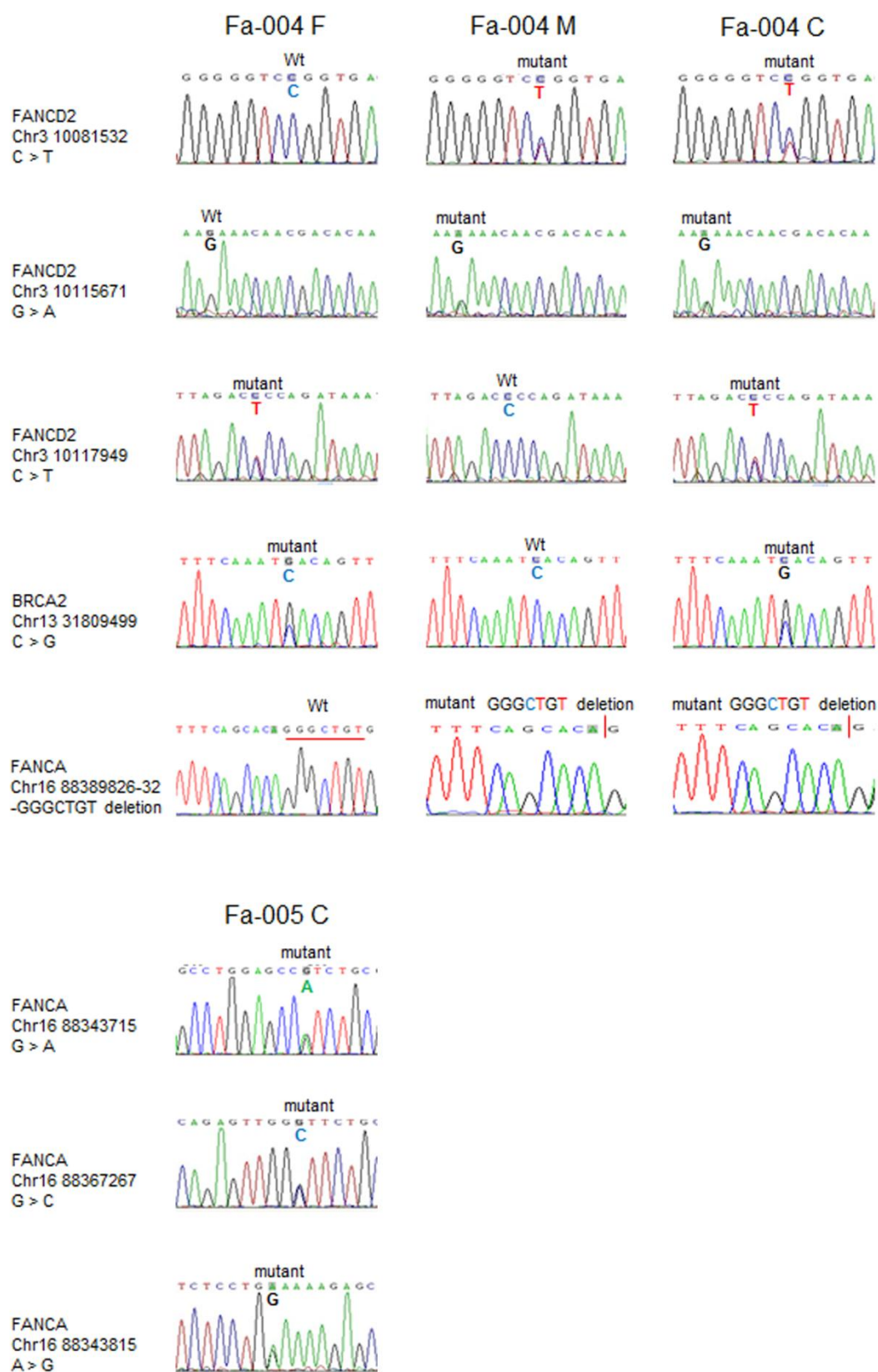

Supplement: Additional file 2: Table S1 — Mitomycin C chromosome fragility test results. [file 1755-8794-7-24-S2.pdf]

**Table S4 MAF, SIFT and Polyphen-2 analysis of FA mutations**


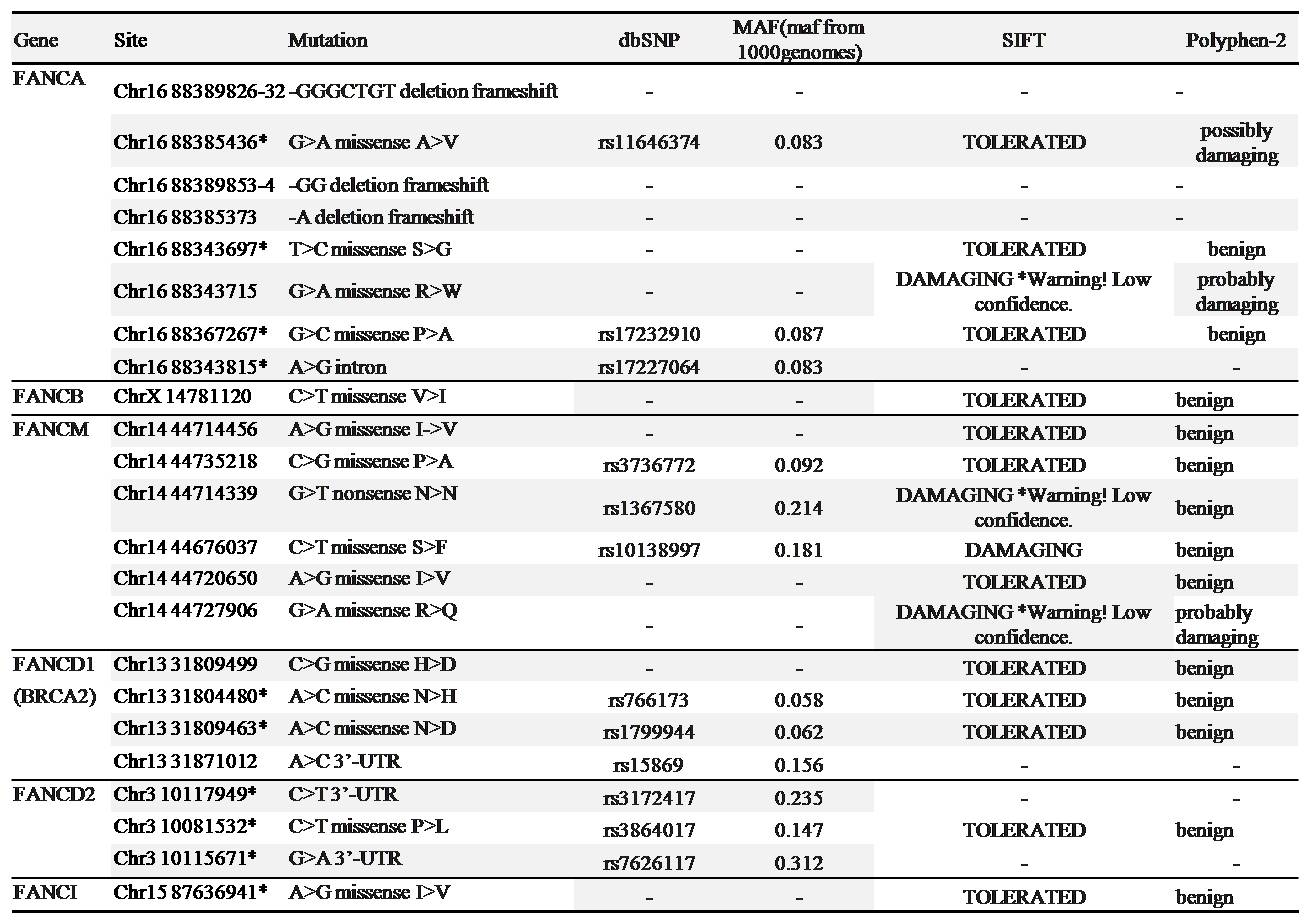

Supplement: Additional file 6: Table S4 — MAF, SIFT and Polyphen-2 analysis of FA mutations. [file 1755-8794-7-24-S6.doc]
