# Supplementary material for: Whole exome sequencing reveals concomitant mutations of multiple FA genes in individual Fanconi anemia patients
Source: BMC Med Genomics. 2014 May 15;7:24. doi: 10.1186/1755-8794-7-24 (PMC4038598; doi:10.1186/1755-8794-7-24)
Supplement: Additional file 3: Table S2 — Single-cell gel electrophoresis test results. [file 1755-8794-7-24-S3.doc]

**Table S1.** Mitomycin C chromosome fragility test results

| **MMC (ng/ml)** | **0** | **40** | **80** |
| --- | --- | --- | --- |
| **Abnormality rate (%)** |  |  |  |
| **WT** | 4 | 7 | 15 |
| **Fa-001** | 4 | 8 | 32 |
| **Fa-002** | 4 | 8 | 33 |
| **Fa-003** | 4 | 8 | 34 |
| **Fa-004** | 4 | 9 | 34 |
| **Fa-005** | 4 | 10 | 40 |
