# Supplementary material for: Whole exome sequencing reveals concomitant mutations of multiple FA genes in individual Fanconi anemia patients
Source: BMC Med Genomics. 2014 May 15;7:24. doi: 10.1186/1755-8794-7-24 (PMC4038598; doi:10.1186/1755-8794-7-24)
Supplement: Additional file 4: Table S3 — Karyotype and bone marrow cellularity of FA patients. [file 1755-8794-7-24-S4.doc]

**Table S2.** Single-cell gel electrophoresis test results

|  | **Head DNA (%)** | **Tail DNA (%)** | **Tail length (pix)** | **Comet length (pix)** | **Tail moment** | **Olive tail moment** | **Comet cell rate*** |
| --- | --- | --- | --- | --- | --- | --- | --- |
| **WT** | 98.82 | 1.18 | 3.68 | 42.36 | 0.05 | 0.19 | <10% |
| **Fa-001** | 69.21 | 30.79 | 39.91 | 131.18 | 6.69 | 5.51 | 69% |
| **Fa-002** | 69.94 | 30.06 | 42.84 | 136.89 | 7.98 | 6.45 | 62% |
| **Fa-003** | 70.82 | 29.18 | 52.63 | 129.37 | 12.07 | 5.39 | 59% |
| **Fa-004** | 70.14 | 29.86 | 69.19 | 121.13 | 12.29 | 10.19 | 57% |
| **Fa-005** | 68.72 | 31.28 | 72.29 | 142.06 | 8.64 | 9.87 | 92% |

***** t student test, p < 0.01. The comet cell rates showed statistically significant differences between patients and wild type samples.
