# Supplementary material for: Whole exome sequencing reveals concomitant mutations of multiple FA genes in individual Fanconi anemia patients
Source: BMC Med Genomics. 2014 May 15;7:24. doi: 10.1186/1755-8794-7-24 (PMC4038598; doi:10.1186/1755-8794-7-24)
Supplement: Additional file 5: Figure S2 — FA gene re-sequencing results of the FA patients and their parents. [file 1755-8794-7-24-S5.doc]

**Table S3. Karyotype and bone marrow cellularity of FA patients**

|  | 001 | 002 | 003 | 004 | 005 |
| --- | --- | --- | --- | --- | --- |
| Karyotypes | 46,XY[20] | 46,XY[18] | 46,XY[16] | 46,XX[19] | 46,XX[20] |
| Bone marrow cellularity | hypoplasia | hypoplasia | normal | normal | normal |
| Myelodysplasia | no | no | no | no | nuclear/cytoplasmic dyssynchrony |

**Note: The karyotypes of the five patients were normal.** The bone marrow cellularity levels of patient 001 and 002 are **hypoplastic,** while patient 003, 004 and 005 were **normal**. The bone marrow smear test results were all from local hospitals, and all of the patients had 1- to 6-month histories before admission to our hospital. Because the bone marrow cellularity results must be interpreted in the context of the peripheral blood counts of our patients, patients 003, 004 and 005 were diagnosed with bone marrow failure ranging from mild to severe, depending on their PB counts (Fanconi Anemia: Guidelines for clinical care consensus conference. Chicago, IL. April 10 and 11, 2008/Fanconi anemia: Guidelines for diagnosis and management, Chapter 3: 53).
